# Matrix Metallopeptidase-2 Gene rs2287074 Polymorphism is Associated with Brick Tea Skeletal Fluorosis in Tibetans and Kazaks, China

**DOI:** 10.1038/srep40086

**Published:** 2017-01-12

**Authors:** Junrui Pei, Bingyun Li, Yang Liu, Xiaona Liu, Mang Li, Yanru Chu, Qing Yang, Wei Jiang, Fuxun Chen, Gottfried M. Darko, Yanmei Yang, Yanhui Gao

**Affiliations:** 1 Center for Endemic Disease Control, Chinese Center for Disease Control and Prevention, Harbin Medical University, Harbin 150081, Heilongjiang Province, China; 2 Key Lab of Etiology and Epidemiology, Education Bureau of Heilongjiang Province & Ministry of Health (23618504), Harbin Medical University, Harbin 150081, Heilongjiang Province, China

## Abstract

Brick tea skeletal fluorosis is still a public health issue in the north-western area of China. However its pathogenesis remains unknown. Our previous study reveals that the severity of skeletal fluorosis in Tibetans is more serious than that in Kazaks, although they have similar fluoride exposure, suggesting the onset of brick tea type skeletal fluorosis might be genetically influenced. Here we show that MMP-2 rs2287074 SNP (G/A), but not rs243865, was associated with Brick tea type fluorosis in Tibetans and Kazaks, China. The trend test reveals a decline in probability for skeletal fluorosis with increasing number of A alleles in Tibetans. After controlling potential confounders, AA genotype had about 80 percent lower probability of developing skeletal fluorosis than GG genotype in Tibetans (odds ratio = 0.174, 95% CI: 0.053, 0.575), and approximately 53 percent lower probability in Kazaks (odds ratio = 0.462, 95% CI: 0.214, 0.996). A meta-analysis shows that the AA genotype had approximately 63 percent lower odds (odds ratio = 0.373, 95% CI: 0.202, 0.689) compared with GG genotype within the two ethnicities. A significant correlation was also found between the genotype of MMP2 rs2287074 and skeletal fluorosis severity. Therefore, the A allele of MMP2 rs2287074 could be a protective factor for brick tea skeletal fluorosis.

Brick tea type fluorosis (BTF), the clinical manifestation characterized by dental fluorosis and skeletal fluorosis, is caused by habitual consumption of large volumes of brick tea with high fluoride. A national epidemiological survey conducted by our group shows that BTF is mainly found in remote western and northern border provinces in China, including Tibet, Inner Mongolia, Qinghai, Sinkiang, Sichuan, Gansu, Ningxia and Yunnan^1^. It is predominant amongst minorities, such as Tibetans, Kazaks, Mongolians, Uighurs, and others who are habitual consumer of tea, milk tea, buttered tea and zanba which are all made of brick tea with high fluoride^1^,^2^. It is estimated that about 13 million people are exposed to the risk of BTF (data not published). The prevalence of brick tea skeletal fluorosis is above 30% in some areas of the eight listed provinces, in China^1^. Skeletal fluorosis, the most serious problem of BTF, is characterized by osteosclerosis, calcification of soft tissue around the bone, the acceleration of bone turnover, osteoporosis, osteomalacia and bone joint degeneration. Patients with skeletal fluorosis experience bone joints pain, physical limitations, and in extreme cases disability^3^. Till date, BTF is still considered a severe public health issue in parts of China, because it is impossible to alter the brick-tea habitual consumption among these minorities.

Skeletal fluorosis is known to be a result of excessive fluoride intake; however, our recent researches suggest that the pathogenesis is probably multifactorial, due to a combination of environmental and genetic risk factors. Our epidemiological studies show that skeletal fluorosis in Tibetans is more serious than other minorities, suggesting that the prevalence rate of brick tea type skeletal fluorosis might be genetically influenced^3^,^4^. We also found that fluoride exposure is similar between Tibetans and Kazaks, however the severity of skeletal fluorosis in Tibetans is more advanced than in Kazaks^3^. The results suggest that the prevalence rate of brick tea type skeletal fluorosis might be genetically linked. Our findings and that of another group have shown gene polymorphism in GSTP1^3^ and myeloperoxidase gene^5^ might be associated with skeletal fluorosis, suggesting that some genetic factors that play a role in the pathogenesis of skeletal fluorosis. Unfortunately, they do not account for all cases. Hence it is important to identify additional candidate genes that might influence the risk of disease for the development of more effective preventive and treatment measures.

Skeletal fluorosis is a chronic metabolic bone and joint disease. There are growing evidences that MMP-2 plays an important role in the pathogenesis of bone metabolism disorders. MMP2, also known as gelatinase A, is a membrane-bound protein that is responsible for extracellular matrix degradation^6^. It is primarily expressed and secreted by osteoblasts and osteocytes in bone tissue^7^,^8^. Several research papers have shown that MMP-2 deficient mice display bone metabolism imbalance including loss of bone volume, mineralization abnormalities and joint erosion^8^,^9^,^10^. Osteoblast and osteoclast numbers were significantly decreased and their differentiation was restricted in MMP-2 deficient mice^9^. Bone marrow cells from MMP-2^−/−^ mice are unable to effectively support osteoblast and osteoclast growth and differentiation in culture^9^. Clinical studies revealed that MMP-2 expression has been shown to be up-regulated in human degenerative disc and the implant-prosthetic rehabilitation^11^,^12^. Other studies have shown a significant negative correlation between serum concentration of MMP-2 and bone mineral density (BMD) in postmenopausal Chinese women^13^, and MMP-2 could be used to evaluate bone remodelling and bone turnover^13^,^14^. Alternatively, the MMP-2 rs243865 single nucleotide polymorphism (SNP) (C/T) has been well characterized, and the C allele is associated with increased gene expression^15^. This SNP of MMP-2 has been shown to be associated with vertebral fracture^16^. The other SNP of MMP-2, rs2287074, has been suggested to be associated with stroke, obesity and maculopathy^17^,^18^,^19^. These studies suggest that MMP-2 plays a pivotal role in skeletal development and bone cell growth and proliferation, and that, some genetic viariants may be associated with the increased or decreased susceptibility to some diseases.

Recent studies have revealed that fluoride can affect the expression of MMP-2 in cell and animal experiment^20^,^21^. An epidemiological survey reveals that an increase of MMP-2 is positively correlated with fluoride exposure and the severity of dental fluorosis in adults^22^. These results suggest that MMP-2 might be a contributory factor for the onset of fluorosis. Recent observations points to a relationship between MMP-2 SNPs and the risk of diseases^16^,^17^,^18^,^19^, and ethnicity differences in which genetic susceptibility to diseases have been proven^23^,^24^. Therefore, we hypothesize that MMP-2 SNPs may be associated with the ethnic difference of skeletal fluorosis between Tibetans and Kazaks who have similar fluoride intake. In our study, two SNPs in the MMP-2 gene (rs243865 and rs2287074) were investigated for association with skeletal fluorosis. The statistical analyses show a significant lower OR in skeletal fluorosis associated with MMP-2 rs2287074 allele A. MMP-2 rs2287074 was also correlated with skeletal fluorosis severity, and the presence of A allele in Kazaks were siginificantly higher than that in Tibetans. The A allele of MMP2 rs2287074 was a protective factor for brick tea type skeletal fluorosis, which may be the reason for the differences in the severity of skeletal fluorosis between Tibetans and Kazaks.

## Materials and Methods

### Subjects

A cross sectional study was conducted in seven villages from two provinces (Qinghai, Sinkiang), People’s Republic of China, where brick-tea type fluorosis is prevalent from July to August 2012. The brick-tea type fluorosis village was identified as one in which people aged 16 years or older took the tea fluoride above 3.5 mg daily, and had skeletal fluorosis confirmed by X-ray (GB17018–2011, China). The subjects enrolled in this cross sectional study were older than 16 years, born and bred in the named villages. The subjects were investigated using a questionnaire which was designed to obtain name, address, sex, age, nationality, disposable income per capita, calcium (Ca) supplement, past medical history, personal history of brick tea consumption, and the volume of brick tea consumed daily. The face-to-face interview was performed by well-trained staff. Every subject received clinical examination which included physical examination and X-ray diagnosis (Beijing Longsafe Imaging Technology Co., Beijing City, China). In addition, brick tea water, blood and urine was collected from each participant.

### Diagnosis of skeletal fluorosis

The radiograph of forearm, shank and pelvic of each participant was used to evaluate the skeletal fluorosis. Skeletal fluorosis was diagnosed and classified according to the Diagnostic Criteria of Endemic Skeletal Fluorosis (WS192–2008, China) as previously described^3^. Briefly, an X-ray of a skeletal fluorosis patient shows osteosclerosis, soft tissue calcification around the bone, acceleration of bone turnover, osteoporosis, osteomalacia and joint degeneration. Based on the results of the X-ray, skeletal fluorosis could be classified into three gradations: mild, moderate and severe.

### Fluoride analysis

The brick tea water sample or urine sample was stored at −20 °C until analyse. The fluoride content of tea water was detected by F-ion selective electrode (Yingke Crystal Materials Company) with a national standardized method of China (GB19965–2005, China). All the samples were assayed twice, and the means of the two results were used as the final fluoride concentration. The urine fluoride was assessed by the standard method for urine fluoride (WS/T 89–2015, China).

### Genotyping methods

MMP-2 rs243865(C/T) and rs2287074 (A/G) are located in promoter region and in codon 460 of exon 9, respectively. Genomic DNA was extracted from whole blood with DNA extraction kit (Axygen Biosciences, Union City, USA). The DNA concentration was determined by TU1901 Spectrophotometry (Purkinje General Company, Beijing City, China) to ensure the DNA concentration was greater than 20 μg/ml. The extracted genomic DNA was stored at −80 °C. All gene sequencing were performed by the Shanghai Fenglin Clinical Laboratory Company (http://www.fenglinlab.com/index.asp) using the Sequenom MassARRAY system (Sequenom, Inc., San Diego, CA, USA).

The primer sequences of MMP-2 rs243865 are:

forward-5′- ACGTTGGATGTGTTCCCTAAAACATTCCCC-3′,

reverse-5′-ACGTTGGATGAGTGACTTCTGAGCTGAGAC-3′, extended-5′-TTCCCCACCCAGCACTC -3′.

The primer sequences of MMP-2 rs2287074 are:

forward-5′-ACGTTGGATGTCTAAGGTCAGGTGTTCTCC-3′,

reverse-5′-ACGTTGGATGTTGCAGATCTCAGGAGTGAC-3′,

extended-5′-GGCACCGGCCCCACCCCCAC-3′.

As a control for the sequencing, blinded blood duplicates were used.

### Potential confounders

Except fluoride intake, age, gender, altitude, occupation, calcium supplement and income were also associated with skeletal fluorosis^4^,^25^,^26^,^27^,^28^,^29^. Fluoride intake was calculated according to the fluoride content of tea water, personal history of brick tea consumption and the volume of brick tea consumed daily. Age, gender and Ca supplement were investigated by a questionaire. Occupation of the two ethnicities included herdsman, farmer, teacher, monk, public servants and freelancer. They were classfied into three groups: herdsman, farmer and others, because there were relatively fewer teachers, monks, public servants and freelancers. Disposable income per capita was determined by asking subjects and divided into four groups: income ≤ 1000 RMB, 1000 RMB < income ≤ 2000 RMB, 2000 RMB < income ≤ 3000 RMB and income >3000 RMB. The altitude of investigated sites was collected from local government.

### Ethical approval and informed consent

The study was approved by the Ethical Review Board of Harbin Medical University (HMUIRB20120021). All participants signed informed consent, and written informed consent was obtained from the guardians of minors. No specific permits were required for the locations or activities associated with the brick-tea water sample collection in this field study. The locations were not privately owned or protected in any way and this field study did not involve endangered or protected species. The methods were carried out in accordance with the approved guidelines.

### Statistical analysis

Testing of Hardy-Weinberg equilibrium was performed stratified by ethnicity using likelihood ratio tests. Comparisons of variables by disease status were made with χ^2^ tests, T-test and Mann-Whitney U tests. The 2 × 2 table analysis was performed with Pearson Chi-square tests. The contingency table analysis was performed with Likehood-Ratio test. The table analysis was performed with Fisher’s Exact test when the expected value of more than one cell of tables was less than 5. The association of genotype and skeletal fluorosis was assessed by Cochran-Armitage trend test. The trend test was performed with R software. Odds Ratios (OR) and corresponding 95% confidence intervals (CI) were calculated for skeletal fluorosis risk by bivariate logistic regression. A fixed effects meta-analysis model was used to merge ORs of the two ethnicities. The association between MMP-2 rs2287074 and skeletal fluorosis was tested by the additive models. I^2^ > 50% indicates significant heterogeneity. The overall OR with 95% CI was calculated using the fixed effects model, and stratified by age (age ≤ 45, 45 < age ≤ 65 and age > 65) because it was the common confounding factor in the two ethnicities. Correlation of two MMP-2 SNPs with skeletal fluorosis was assessed by Spearman test. P < 0.05 was considered statistically significant. All statistical analyses were performed with STATA (STATA, College Station, TX, Version 12.0). The data of this study had been provided in Supplementary information.

## Results

A total of 598 subjects were enrolled in this study. 221 subjects were diagnosed with skeletal fluorosis, and the prevalence of skeletal fluorosis was 37.0%. The medians of fluoride intake and urine fluoride were 5.985 mg/L and 2.755 mg/L in enrolled participants, respectively. The prevalence of skeletal fluorosis and fluoride exposure between Tibetan and Kazak were shown in Table 1. Compared to Kazaks, Tibetans had a higher prevalence of developing skeletal fluorosis and lower urine fluoride concentration, but this was not significantly different (Pearson Chi-square = 2.422, p = 0.12; Z = 5.359, p < 0.001). However, the proportions of moderate and severe skeletal fluorosis in Tibetans were significantly higher than that in Kazaks (Likehood Ratio = 24.145, p < 0.001), and fluoride intake was not significantly different between Tibetans and Kazaks (Z = 0.294, p = 0.435). Above results demonstrated that skeletal fluorosis was a predominant disease, and fluoride exposure was higher compared with the standard of fluoride intake (3.5 mg) (WS/T 87–1996) and urine fluoride concentration (1.6 mg/L) (WS/T 256–2005, China) in the two ethnicities.

The descriptive analysis of potential risk factors between skeletal fluorosis cases and controls were presented and stratified by ethnicity in Table 2. Fluoride intake and urine fluoride in the Tibetan cases were both significantly higher than those in Tibetan controls (Z = 2.354, p = 0.019; Z = 2.477, p = 0.013, respectively), but there were no significant difference in Kazak participants (Z = −1.614, p = 0.106; Z = −0.631, p = 0.528, respectively). The cases were significantly older than the controls in the two ethnicities (t = 7.078, p  < 0.01 in Tibetans; t = 2.034, p = 0.043 in Kazaks, respectively). The difference of gender proportion between the cases and the controls was detected in Tibetans (Pearson Chi-square = 5.595, p = 0.018), but not found in Kazaks (Pearson Chi-square = 3.373, p = 0.066). The occupational proportions between the cases and the controls were not different in the two ethnicities (Fisher’s Chi-square = 1.012, p = 0.687 in Tibetans; Fisher’s chi-square = 0.692, p = 0.909 in Kazaks). Above 80% of Tibetans were herdsman, and 90% of Kazaks were farmers in this study. The income between the cases and the controls was not statistically different in the two ethnicities (Likehood Ratio = 0.892, p = 0.827 in Tibetans; Likehood Ratio = 1.326, p = 0.723 in Kazaks). The frequency of Ca supplement between the cases and the controls was not significantly different in Tibetans and Kazaks (p > 0.05). Participants suffering from skeletal fluorosis lived in higher altitudes than the controls among Tibetans (Z = 2.587, p = 0.01), but this altitude was not different in Kazaks (Z = 0.379, p = 0.705). From the results, some confounding factors were identified in the two ethnicities: fluoride intake, urine fluoride, gender, age and altitude were identified among Tibetans, and age was a confounding factor for Kazaks. Age was the common confounding factor among the two ethnicities.

The association between two SNPs of MMP-2 gene and skeletal fluorosis was investigated in the two ethnicities. The hypothesis of Hardy-Weinberg equilibrium could not be rejected for each of the two SNPs. Analysis of the genotype distribution shows that the genotype frequency of MMP-2 rs243865 between the cases and the controls were not statistically different in the two ethnicities (Likehood Ratio = 1.270, p = 0.530 in Tibetans; Likehood Ratio = 0.533, p = 0.766 in Kazaks). However, the genotype frequency of MMP-2 rs2287074 differed significantly between the cases and controls in Tibetans (Likehood Ratio = 8.704, p = 0.013), but not significantly in Kazaks (Likehood Ratio = 3.738, p = 0.154). The Cochran Armitage trend test and the logistic regression analysis were further performed to estimate the association between MMP-2 rs2287074 and the risk of skeletal fluorosis. The trend test revealed a declining probability for skeletal fluorosis with increasing number of A allele in Tibetans (Chi-square = 7.196, p = 0.007). In the logistic regression analysis, we adjusted for fluoride intake, urine fluoride, age, gender and altitude in Tibetans, and adjusted for age in Kazaks. After controlling potential confounders, there were significantly lower odds of skeletal fluorosis in participants with AA homozygotes. AA genotype had about 80 percent lower odds of having skeletal fluorosis than GG genotype in Tibetans (Odds Ratio = 0.174, 95% CI: 0.053, 0.575), and approximately 53 percent lower odds in Kazaks (Odds Ratio = 0.462, 95% CI: 0.214, 0.996) (Table 3).

A meta-analysis was conducted to assess the association between MMP-2 rs2287074 and skeletal fluorosis risk in the two ethnicities. We defined the inheritance model by treating allele A as the ‘risk’ allele. The meta-analysis was stratified by age which was the common confounding factor in the two ethnicities. Table 4 shows that the AG genotype were not significantly associated with the risk of skeletal fluorosis compared with the GG genotype. However, the AA genotype was associated with a significant decrease in risk of skeletal fluorosis compared with GG genotype (Odds Ratio = 0.373, 95% CI: 0.202, 0.689). The stratified analysis shown that the A allele was not associated with the risk of skeletal fluorosis in subjects with age ≤ 45. However the A allele was significantly associated with skeletal fluorosis in subjects with 45 < age ≤ 65. The AA genotype was significantly associated with a decrease in risk of skeletal fluorosis compared with GG genotype in subjects aged 45 years and above (Odds Ratio = 0.403, 95% CI: 0.191, 0.850; Odds Ratio = 0.168, 95% CI: 0.031, 0.902, respectively).

Furthermore, a significant correlation between the allele of MMP2 rs2287074 and skeletal fluorosis severity was found in these participants (r = −0.151, p < 0.01). The correlation was separately detected in Tibetans and Kazaks (r = −0.148, p < 0.01; r = −0.122, p = 0.038). Subjects with AG or AA genotypes had less severity skeletal fluorosis. Particularly, there was only one moderate, and none severe skeletal fluorosis among subjects with AA genotype (Table 5).

## Discussion

Skeletal fluorosis is a chronic metabolic bone disease, which is caused by excessive fluoride intake^30^. Bone matrix degradation is a key step in bone metabolism in which MMPs play an important role. MMPs, zinc-dependent proteases, are able to degrade bone matrix proteins such as collagen and elastin^31^. Matrix Metallopeptidase-2 (MMP-2), isolated from the culture media of rheumatoid arthritis synovial tissue^32^, is important for extracellular matrix degradation^33^. It has proteolytic activity against components of the basement membrane, preferentially cleaving collagen types I, IV, V, VII, and XI and gelatin^6^,^34^,^35^. Recently, the association between MMP-2 and fluorosis has been explored^22^. An *in vitro* study shown that MMP-2 activity of human saliva significantly inhibited by excessive fluoride (above 50 mg/L)^20^. However, an increase of MMP-2 expression in gingival connective tissue and periodontal ligament was found in rabbits treated with 40 mg/L of fluoride^21^. In a word, though the effect of fluoride on MMP-2 remains assumptive, excessive fluoride exposure could alter MMP-2 activity and expression. An investigation in human population also shown that a positive dose-response relationship between MMP-2 and fluoride exposure and the severity of dental fluorosis was separately observed in adults^22^, suggesting that MMP-2 could be involved in the pathogenesis of skeletal fluorosis.

Previous researches have shown that MMP-2 rs243865 was associated with the increased expression of MMP-2^15^, and white women with CT genotype of MMP-2 rs243865 had a lower rate of vertebral fracture in USA^16^. The other synonymous SNP of MMP-2 rs2287074 has been found for associations with stroke, obesity and maculopathy^17^,^18^,^19^, but has no association with fracture risk and BMD^16^. This is the first epidemiologic study that evaluates the association between MMP-2 polymorphisms and skeletal fluorosis. In our study, the MMP-2 rs2287074 was associated with significantly lower odds of skeletal fluorosis, but the association between MMP-2 rs243865 and skeletal fluorosis was not seen. After controlling for potential confounders, the AA genotype of MMP-2 rs2287074 was separately associated with significantly lower odds of skeletal fluorosis in Tibetans and Kazaks. We further performed a meta-analysis to estimate the overall OR of the two ethnicities and OR stratified by age. The AA genotype of MMP-2 rs2287074 remained significantly associated with lower odds of skeletal fluorosis in all subjects. It has been reported that the prevalence of skeletal fluorosis was significantly associated with age^36^, and it was the common confounding factor in the two ethnicities. So we investigated the potential interactions between MMP2 rs2287074 and age. This protective effect of AA genotype of MMP2 rs2287074 was detected only in subjects with age > 45, but not in subjects with age ≤ 45. In present study, we also found that the prevalence of skeletal fluorosis in subjects with age ≤ 45 was lowest than two other older groups. Therefore we speculate large sample is required to get difference while the prevalence rate of fluorosis is low in the subjects with age ≤ 45. Also, there is a significant correlation of the MMP-2 rs2287074 variant with skeletal fluorosis severity, and subjects with the AA genotype had less risk to suffer from moderate and severe skeletal fluorosis in the two ethnicities. These results suggest that MMP-2 rs2287074 was not only associated with skeletal fluorosis, but also correlated with skeletal fluorosis severity. The more severe of skeletal fluorosis in Tibetans might be associated with the lower frequency of MMP-2 rs2287074 A allele.

Although we found that genotype AA of MMP2 rs2287074 was a protective factor for brick tea skeletal fluorosis, the specific function of the MMP2 rs2287074 SNP in skeletal fluorosis is unknown. This SNP is synonymous, resulting in the same amino acid (threonine) at codon 460 regardless of the allele present. Although this kind of variant does not appear to create a new splice site or alter an existing one, it has been shown that variation at synonymous sites could alter RNA secondary structures, affecting RNA stability, which result in the changes of protein expression and function^37^,^38^,^39^. Nevertheless, the specific functionality of MMP-2 rs2287074 in skeletal fluorosis needs to be further clarified.

There were several limitations in this study. Sample size of our study was too small to affect observed power, such as the frequency of MMP-2 rs 2287074 AA genotype was fewer in the two ethnic groups. The association could be as a result of confounding by unknown factors. Moreover, skeletal fluorosis is complex disease and it is likely that several genes and/or polymorphic sites influence its malformations. Thus, the nature of the association of this polymorphism with skeletal fluorosis needs to be further clarified.

## Conclusions

In summary, in this study, we found there was significantly lower chances of skeletal fluorosis developing in subjects with the A allele of MMP2 rs2287074. The AA genotype of MMP2 rs2287074 was also shown to be inversely associated with skeletal fluorosis severity. The differences in skeletal fluorosis severity between Tibetans and Kazaks are possibly induced by the different frequency of MMP2 rs2287074 genotypes. These results need to be replicated in future studies, particularly with sufficiently larger sample sizes, to confirm this association in other ethnicities. In addition, the direct or indirect role of SNPs in skeletal fluorosis pathogenesis should be further investigated.

## Additional Information

**How to cite this article**: Pei, J. *et al*. Matrix Metallopeptidase-2 Gene rs2287074 Polymorphism is Associated with Brick Tea Skeletal fluorosis in Tibetans and Kazaks, China. *Sci. Rep.* **7**, 40086; doi: 10.1038/srep40086 (2017).

**Publisher's note:** Springer Nature remains neutral with regard to jurisdictional claims in published maps and institutional affiliations.

## Supplementary Material

Supplementary Information

## Figures and Tables

**Table 1:** The prevalence of skeletal fluorosis and fluoride exposure between Tibetans and Kazaks.

Viariable	All subjects	Tibetans	Kazaks
	n (%)	n (%)	n (%)
	n = 598	n = 308	n = 290
Fluoride intake (mg)	5.985(3.749, 10.102)	5.895(3.797, 9.495)	6.142(3.642, 10.759)
Urine fluoride (mg/L)	2.755(1.763, 4.138)	2.359(1.575, 3.628)	3.166(2.060, 4.576)*
Skeletal fluorosis			
cases	221(36.96%)	123(39.9%)	98(33.8%)
controls	377(63.04%)	185(60.1%)	192(66.2%)
Skeletal fluorosis severity			
no	377(63.04%)	185(60.06%)	192(66.21%)
mild	150(25.08%)	68(22.08%)	82(28.28%)
moderate	43(7.19%)	32(10.39%)	11(3.79%)*
severe	28(4.68%)	23(7.47%)	5(1.72%)*

Note: Fluoride intake and urine fluoride were represented as median (P25, P75) and tested by Mann-Whitney U test. Z was 0.294 and 5.359, respectively; the comparison of skeletal fluorosis and skeletal fluorosis severity between the two ethnic groups were performed by Likehood Ratio test. Likehood Ratio was 2.415 and 24.145, respectively.*, compare with Tibetans, P < 0.05.

**Table 2:** Association of viariables with skeletal fluorosis was evaluated by bivaiate logistic regression model.

Variables	Tibetans			Kazaks		
	Cases (n%)	Controls (n%)	*P*	Cases (n%)	Controls (n%)	*P*
	n = 123	n = 185		n = 98	n = 192	
Fluoride intake (mg)	7.051(4.371, 10.968)	5.305(3.727, 8.617)	**0.019**	5.201(3.501, 9.073)	6.51(3.849, 11.784)	0.106
Urine Fluoride (mg/L)	2.636(1.801, 4.138)	2.172(1.406, 3.512)	**0.013**	3.093(1.844, 4.495)	3.215(2.084, 4.585)	0.528
Age (years)	59 ± 12	49 ± 12	**<0.001**	55 ± 10	52 ± 12	**0.043**
Gender						
female	59(48%)	114(61.6%)	**0.018**	54(55.1%)	127(66.1%)	0.066
male	64(52%)	71(38.4%)		44(44.9%)	65(33.9%)	
Occupation						
herdsman	101(82.1%)	152(82.2%)	0.687	1(1.0%)	1(0.5%)	0.909
farmer	0(0%)	2(1.1%)		92(93.9%)	179(93.2%)	
other	22(17.9%)	31(16.8%)		5(5.1%)	12(6.3%)	
Disposable income per capita (RMB)						
income ≤ 1000	56(45.6%)	92(49.73%)	0.827	4(4.1%)	9(4.7%)	0.723
1000 < income ≤ 2000	39(31.7%)	50(27.03%)		32(32.6%)	68(35.4%)	
2000 < income ≤ 3000	9(7.3%)	15(8.11%)		28(28.6%)	43(22.4%)	
income > 3000	19(15.4%)	28(15.14%)		34(34.7%)	72(37.5%)	
Ca supplement						
with	2(1.6%)	1(0.5%)	0.566	6(6.1%)	26(13.5%)	0.056
without	121(98.4%)	184(99.5%)		92(93.9%)	166(86.5%)	
Altitude (metre)	3985(3929, 3985)	3929(3929, 3985)	**0.01**	571(552, 571)	571(552, 571)	0.705
MMP-2rs243865						
CC	88(71.5%)	136(73.5%)	0.53	59(60.2%)	107(55.7%)	0.766
CT	32(26%)	41(22.2%)		34(34.7%)	74(38.5%)	
TT	3(2.4%)	8(4.3%)		5(5.1%)	11(5.7%)	
MMP-2rs2287074						
GG	66(53.7%)	77(41.6%)	**0.013**	39(39.8%)	61(31.8%)	0.154
AG	53(43.1%)	88(47.6%)		47(48.0%)	92(47.9%)	
AA	4(3.3%)	20(10.8%)		12(12.2%)	39(20.3%)	

Note: Age was represented as Mean ± SD; Fluoride intake, urine fluoride and altitude were represented as median (P25, P75). Bold font, p < 0.05.

**Table 3:** Association of MMP-2 Rs2287074 with skeletal fluorosis in Tibetans and Kazaks.

Genotype	Tibetans (n = 308)			Kazaks (n = 290)		
	Cases	Controls	OR (95% CI)*	Cases	Controls	OR (95% CI)^#^
	n (%)	n (%)		n (%)	n (%)	
	n = 123	n = 185		n = 98	n = 192	
GG	66(53.7%)	77(41.6%)	1(Reference)	39(39.8%)	61(31.8%)	1(Reference)
AG	53(43.1%)	88(47.6%)	0.588(0.343, 1.007)	47(48%)	92(47.9%)	0.802(0.468, 1.373)
AA	4(3.3%)	20(10.8%)	**0.174(0.053, 0.575)**	12(12.2%)	39(20.3%)	**0.462(0.214, 0.996)**

Note: *Adjusted for fluoride intake, urine fluoride, age, gender and altitude. ^#^Adjusted for age. Bold font, p < 0.05.

**Table 4:** Pooled estimates of ORs (95% CI) for the association between MMP-2 Rs2287074 and skeletal fluorosis.

Inheritance model	Cases/controls	OR	95% CI	*P*	I^2^	*P* for heterogeneity*
All subjects						
AG versus GG	221/377	0.741	0.52~1.055	0.096	0%	0.578
AA versus GG		0.373	0.202~0.689	**0.002**		
Age ≤ 45						
AG versus GG	40/144	0.751	0.365~1.544	0.436	0%	0.937
AA versus GG		0.165	0.020~1.339	0.092		
45 < age ≤ 65						
AG versus GG	129/183	0.592	0.362~0.969	**0.037**	31.0%	0.235
AA versus GG		0.403	0.191~0.850	**0.017**		
Age > 65						
AG versus GG	52/50	1.126	0.462~2.744	0.794	0%	0.894
AA versus GG		0.168	0.031~0.902	**0.038**		

**Table 5:** The correlation of MMP-2 Rs2287074 with skeletal fluorosis severity.

Genotype	No	Mild	Moderate	Severe	*r*	P*
	n (%)	n (%)	n (%)	n (%)		
	n = 377	n = 150	n = 43	n = 28		
All subjects						
GG	138(36.6%)	64(42.7%)	26(60.5%)	15(53.6%)	−0.151	<0.01
AG	180(47.7%)	71(47.3%)	16(37.2%)	13(46.4%)		
AA	59(15.6%)	15(10%)	1(2.3%)	0(0%)		
Tibetans						
GG	77(41.6%)	35(51.5%)	19(59.4%)	12(52.2%)	−0.148	<0.01
AG	88(47.6%)	29(42.6%)	13(40.6%)	11(47.8%)		
AA	20(10.8%)	4(5.9%)	0(0%)	0(0%)		
Kazaks						
GG	61(31.8%)	29(35.4%)	7(63.6%)	3(60%)	−0.122	0.038
AG	92(47.9%)	42(51.2%)	3(27.3%)	2(40%)		
AA	39(20.3%)	11(13.4%)	1(9.1%)	0(0%)		
